# Metyrapone in the Management of Severe Hypercortisolism Secondary to Ectopic Cushing’s Syndrome

**DOI:** 10.1155/crie/1796526

**Published:** 2026-09-02

**Authors:** Guy I. Sydney, Spyridon Ntelis, Sachin K. Majumdar

**Affiliations:** ^1^ Section of Endocrinology, Department of Internal Medicine, Yale School of Medicine, New Haven, Connecticut, USA, yale.edu; ^2^ Department of Immunobiology, Yale School of Medicine, New Haven, Connecticut, USA, yale.edu

**Keywords:** ectopic Cushing’s syndrome, hypercortisolism, metyrapone

## Abstract

Ectopic Cushing’s syndrome (ECS) is a rare but important cause of ACTH‐dependent Cushing’s syndrome. Metyrapone, a steroidogenesis inhibitor targeting 11‐β hydroxylase, has shown effectiveness in controlling hypercortisolism. However, little is known about the practical aspects of its initiation, use, and effectiveness in ECS. We present three cases of severe hypercortisolism secondary to ECS managed with metyrapone and report our experience. Case 1: a 56‐year‐old male with high‐grade neuroendocrine carcinoma. Case 2: a 67‐year‐old man with a history of stage IIB non‐small cell lung cancer (NSCLC) with metastatic neuroendocrine carcinoma. Case 3: a 64‐year‐old man with metastatic prostate cancer. Biochemical control was achieved with rapid titration of low doses of daily metyrapone in all three cases. Based on our experience, we recommend an initial dose of 1000–1500 mg of metyrapone daily, in divided doses, with physiologic glucocorticoid replacement to mitigate the risk of adrenal insufficiency. Despite improvements in cortisol levels and stabilization of metabolic derangements, all three patients ultimately transitioned to comfort‐focused care. Given the rarity, heterogeneity, and aggressiveness of this condition, rapid diagnosis and standardized pharmacologic approach are difficult to achieve. More data are needed to guide optimal therapy selection, dosing, and titration in ECS.

**Summary**

• Ectopic Cushing’s syndrome (ECS) is a rare but clinically important subset of ACTH‐dependent Cushing’s syndrome.
• Metyrapone, a steroidogenesis inhibitor targeting 11‐β hydroxylase, can be effective in rapidly reducing cortisol levels in ECS alone or in combination with chemotherapy.
• We recommend initiating 1000–1500 mg of metyrapone daily, in divided doses, with physiologic hydrocortisone while simultaneously discontinuing mineralocorticoid antagonists and closely monitoring electrolytes.
• Management of ECS necessitates a multidisciplinary approach. Timely coordination among specialties is critical for accurate diagnosis, prompt treatment initiation, and alignment of therapeutic strategies with patient‐centered goals.
• More data are needed to guide optimal therapy selection, pharmaceutical dosing, and titration in ECS, underscoring the need for further research and guidelines.

## 1. Introduction

Ectopic Cushing’s syndrome (ECS) results from uncontrolled secretion of ACTH by various neuroendocrine tumors, most commonly lung and pancreatic, and is often associated with metastases at presentation [1]. While the progression of symptoms can be dramatic, the diagnosis may be missed or delayed since profound hypercortisolism can occur without the classical stigmata of Cushing’s syndrome. Manifestations typically include uncontrolled hypertension, hypokalemia, hyperglycemia, and occasionally opportunistic infections. Contrary to Cushing’s disease, catabolic symptoms are more prominent [2]. The prognosis is generally poor, and prompt initiation of treatment is necessary to improve the quality of life and mitigate life‐threatening complications [2]. Pharmacologic control of hypercortisolism is an essential adjunct to tumor‐targeting therapies. Metyrapone, a steroidogenesis inhibitor targeting 11‐β hydroxylase, has shown effectiveness in controlling hypercortisolism, yet little is known about the practical aspects of its initiation, use, and effectiveness in ECS [3–6]. We describe three cases of ECS managed with metyrapone and report our experience with its use.

## 2. Cases Presentation

### 2.1. Case 1

A 56‐year‐old man presented to the hospital with an infected right lower extremity stump. He had recently developed persistent hypertension, severe hyperglycemia out of proportion to prior control, and refractory hypokalemia despite potassium supplementation. Systemic symptoms included an increased appetite, 20‐lb weight gain, fatigue, mood changes, and anxiety over the past year. He denied new bruising, skin thinning, or muscle weakness but reported declining activity and general malaise. The physical exam was notable for left leg edema and an absence of striae and classic features of Cushing’s syndrome. Imaging revealed multiple liver lesions.

### 2.2. Case 2

A 67‐year‐old man with a history of stage IIB non‐small cell lung cancer (NSCLC) and anal squamous cell carcinoma, both in remission for at least 10 years, was referred to endocrinology for evaluation of hypokalemia and hypercortisolism. His original lung cancer had been resected 13 years prior. Recent imaging, performed after a positive QuantiFERON test, revealed a spiculated right lung mass and multiple liver lesions. A liver biopsy demonstrated metastatic neuroendocrine carcinoma, positive for TTF‐1, suggesting a pulmonary origin. He was started on a regimen of carboplatin, etoposide, and atezolizumab. Although initially stable on treatment, he developed progressively worsening hypertension, hyperglycemia, bilateral lower extremity edema, hypokalemia, hypernatremia, and metabolic alkalosis.

At the time of the endocrine evaluation, he reported decreased energy and muscle weakness, describing a decline in his ability to lift weights at the gym by ~50%. He noted recurrent ankle edema. He denied hyperglycemia (reporting an HbA1c typically in the 4% range), facial rounding, weight changes, easy bruising (aside from a thrombocytopenia‐associated episode), or change in height. He was taking a DHEAS‐containing “vitality” supplement and remained on atezolizumab as part of the ongoing oncologic therapy.

### 2.3. Case 3

A 64‐year‐old man with metastatic prostate cancer presented with progressive lower extremity edema, proximal muscle weakness, and intermittent dyspnea, beginning approximately 1 month after initiating flutamide. He denied mood or skin changes, weight loss, or gastrointestinal symptoms. Recent findings included new‐onset hypertension, hypokalemia, hypernatremia, and metabolic alkalosis. He also developed significant transaminitis, which improved with intravenous fluids and discontinuation of flutamide. He noted a possible darkening of the urine. He had not initiated a prescribed diuretic. Notably, no definitive pathology was obtained from his bone biopsy, raising the possibility of tumor evolution.

## 3. Diagnostic Assessment

### 3.1. Case 1

Biochemical evaluation revealed markedly elevated cortisol levels, consistent with ectopic ACTH secretion: morning serum cortisol was >123 µg/dL (measured with an immunoassay in all cases), plasma ACTH was 81 pg/mL, and 24‐h urinary free cortisol (UFC) was 22,528.5 µg/24 h (tandem mass spectrometry, reference range: 4.0–50.0 µg/24 h). A liver biopsy revealed a high‐grade neuroendocrine carcinoma with a Ki‐67 proliferation index of 90%, consistent with an aggressive tumor phenotype. Brain/pituitary magnetic resonance imaging (MRI) demonstrated no evidence of intracranial metastases or pituitary pathology. Computed tomography (CT) identified a 2.9 cm right hilar mass, multiple nodular lesions within the upper lobes of the lungs, and prominent hilar and mediastinal lymphadenopathy, suggestive of thoracic disease involvement.

### 3.2. Case 2

Biochemical evaluation showed progressively increasing plasma ACTH levels (rising from 100 to 210 pg/mL) and inappropriately high morning serum cortisol levels (40, 56, and 67 µg/dL). A 24‐h UFC was markedly elevated at 4345.2 µg/24 h (reference range: 4.0–50.0 µg/24 h). Pituitary protocol MRI revealed no abnormalities. Positron emission tomography (PET) imaging revealed avid nodules in the right upper lobe as well as liver metastases.

### 3.3. Case 3

Morning serum cortisol levels were elevated on repeat testing (53.30 and 67.90 µg/dL), while plasma ACTH was inappropriately within the normal range at 35 pg/mL. A low‐dose dexamethasone suppression test showed failure of cortisol suppression (cortisol: 70.30 µg/dL; dexamethasone level: 684 ng/dL). In addition, 24‐h UFC was markedly elevated at 4452.8 µg/24 h (reference range: 4.0–50.0 µg/24 h). Pituitary protocol MRI revealed no evidence of a pituitary adenoma.

## 4. Treatment, Outcome, and Follow‐Up

### 4.1. Case 1

**Figure 1:**
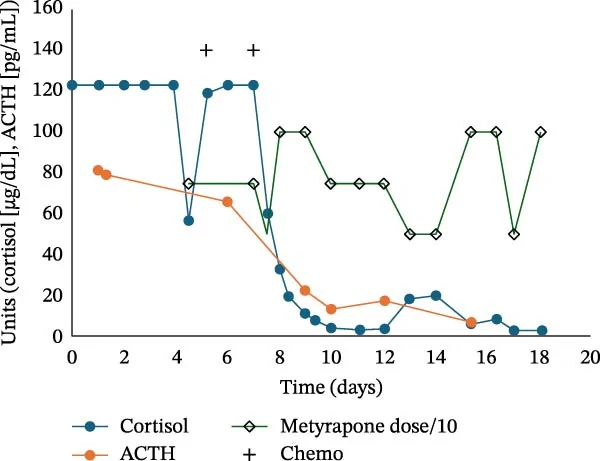
Cortisol, ACTH, and metyrapone dose changes (case 1).

Medical management was initiated at the hospital. Prior to metyrapone introduction, the patient was on spironolactone 300 mg/d, 40 mEq of potassium chloride (KCL) twice daily, amiloride 5 mg/d, amlodipine 10 mg/d, and metoprolol 100 mg twice daily. A “test dose” of 750 mg of metyrapone was given (Figure 1), which lowered cortisol from >123 μg/dL to 57 μg/dL within several hours. Metyrapone was started at 250 mg four times a day on the same day the patient received etoposide (day 1), and by day 3, spironolactone and amiloride were discontinued. Furosemide was continued with KCL 40 mEq/d to treat the edema. Serum potassium levels remained stable. Within 3 days of chemotherapy (etoposide/carboplatin) and metyrapone initiation, cortisol levels declined to 4.6 μg/dL. Dexamethasone (2 mg/d) was started on day 3 of metyrapone treatment and later transitioned to hydrocortisone while continuing metyrapone. Prophylaxis for *Pneumocystis jirovecii* pneumonia was initiated with daily trimethoprim‐sulfamethoxazole. During hospitalization, the initially elevated transaminase levels normalized, and hyperglycemia improved. Throughout the 189 days of follow‐up, biochemical control depended on patient compliance and metyrapone availability. There were instances in which drug delivery was delayed and others in which patient adherence was suboptimal.

### 4.2. Case 2

**Figure 2:**
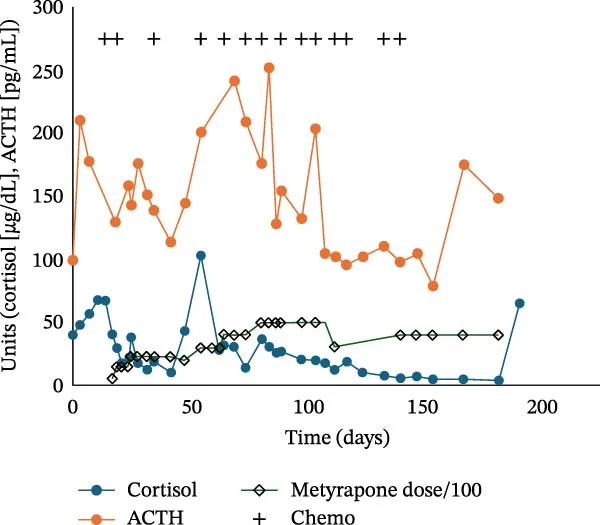
Cortisol, ACTH, and metyrapone dose changes (case 2). A 5‐day course of osilodrostat preceded the initiation of metyrapone.

Medical management was performed in the outpatient setting. Prior to metyrapone, the patient was on 150 mg spironolactone, 15 mg lisinopril, and 30 mEq KCL daily. Osilodrostat and metyrapone were prescribed simultaneously due to concerns regarding insurance coverage and delay in acquisition. Osilodrostat was available first and initiated at 5 mg twice daily for 2 days and 10 mg twice daily for 3 days and subsequently transitioned to metyrapone 500 mg three times daily. Spironolactone and routine potassium supplementation were discontinued on day 7 when metyrapone was increased to 1500 mg/d. A faster and more dramatic decline in cortisol was noted with metyrapone vs. osilodrostat. Hydrocortisone replacement was initiated prior to starting metyrapone given the outpatient setting and concerns for adrenal insufficiency. Potassium levels remained stable, yet replacement was given when needed in setting of periodic furosemide (with spironolactone) to reduce edema. While chemotherapy was given (initially with lurbinectedin and later with paclitaxel), ACTH remained elevated, yet cortisol levels were low, suggesting an effective blockade of cortisol synthesis by metyrapone but ineffective chemotherapy (Figure 2). Over time, blood pressure improved (home readings ~ 130/80 mmHg), hypokalemia resolved, and lower extremity edema gradually decreased. Muscle weakness and fatigue remained notable but stable. A mild elevation of transaminases was steady too.

Ongoing challenges included insurance coverage for metyrapone, necessitating legal advocacy for medication approval, the need for frequent outpatient blood draws during transition, and ultimately limited effectiveness of chemotherapy, all of which together impacted the patient’s quality of life.

### 4.3. Case 3

**Figure 3:**
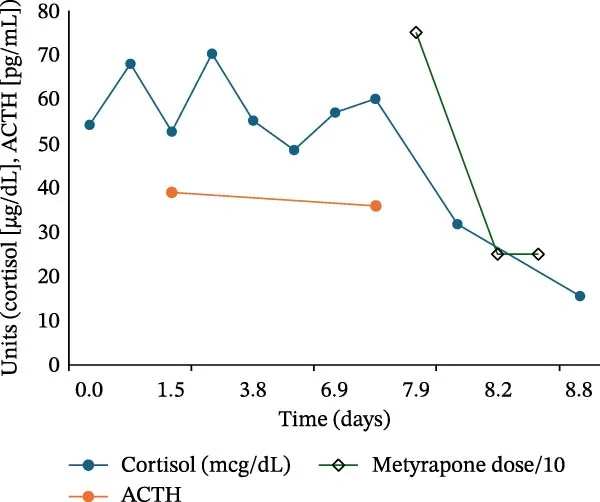
Cortisol, ACTH, and metyrapone dose changes (case 3).

Medical management began in the inpatient setting. Prior to metyrapone, the patient was on spironolactone 300 mg/d, amiloride 5 mg/d, hydrochlorothiazide 25 mg/d, and KCL 40 mEq/d. Metyrapone was initiated at 250 mg four times daily, and by the next day, cortisol levels had declined from 60 μg/dL to 15.5 μg/dL, at which time blood pressure medicines were reduced. Dexamethasone (0.5 mg/d) was started to avoid iatrogenic adrenal insufficiency (Figure 3). Potassium levels were stable and blood pressure improved; yet, given the patient’s preference to pursue hospice care, chemotherapy and surgery were deferred.

## 5. Discussion

In this article, we describe three patients with severe hypercortisolism secondary to ECS who were medically managed with metyrapone, a steroidogenesis inhibitor with a rapid onset of action. In all cases, metyrapone was effective in reducing cortisol levels within hours to days and in achieving biochemical control paralleled by clinical improvements. Notably, we were successful in achieving a biochemical response utilizing rapid titration of low doses of metyrapone (1000–1500 mg daily). The block‐and‐replace strategy—steroidogenesis inhibition with physiologic glucocorticoid replacement—was used to mitigate the risk of adrenal insufficiency, especially as cortisol levels declined. While typically larger doses of metyrapone are suggested for this strategy, our data suggest that lower doses could be utilized, enabling a more manageable safety profile.

We recommend initiating 1000–1500 mg of metyrapone daily, in divided doses, with physiologic hydrocortisone while simultaneously discontinuing mineralocorticoid antagonists and closely monitoring electrolytes. Physiologic glucocorticoid replacement is unlikely to play a significant role in worsening hypercortisolemia and may provide protection against an adrenal crisis. Additionally, this strategy allows for higher initial metyrapone doses and stability in cortisol levels against varying effects of chemotherapy, which is often initiated at the time of diagnosis. In case 1, chemotherapy proved effective, supported by a rapid drop in ACTH, yet without glucocorticoid replacement, an adrenal crisis might have ensued.

Interestingly, in case 2, a relatively low metyrapone dose, 1500 mg daily, appeared superior to a higher osilodrostat dose, 20 mg daily, in reducing hypercortisolism. While contradictory to the efficacy profiles of these agents, it is important to consider that osilodrostat was given prior to metyrapone and may have potentiated the rapid decline in cortisol, and therefore a true comparison of drug efficacy is not possible [7, 8]. These findings do, however, raise the combination of steroidogenesis inhibitors as a potential intriguing strategy, warranting further investigation.

Hypokalemia, alkalosis, and low eosenophil counts were universal findings across all cases. Our patients required potassium repletion and the use of mineralocorticoid receptor antagonists [9]. Hypokalemia, along with metastatic, unresectable malignancy, new‐onset diabetes, and severe hypercortisolism, have been associated with worse prognosis [10]. A rapid lowering of cortisol could simplify medication and supplementation adjustments by achieving earlier electrolyte stability and control. Hospitalization, as seen in two of our cases, could be prudent for metyrapone initiation in patients on complex regimens with high mineralocorticoid and potassium requirements, where frequent electrolyte monitoring and replacement are necessary.

UFC levels were useful in confirming severe hypercortisolism and for therapeutic monitoring, to assess control, even on hydrocortisone, in conjunction with clinical parameters. Given the delay in results, clinical circumstances along with measures such as serum cortisol, ACTH, and dexamethasone suppression testing should be used for the initial diagnosis to avoid delays. ACTH could also be used as a primary tumor marker, while serum and urinary cortisol levels may reflect either treatment of the primary tumor or the inhibition of cortisol synthesis.

While biochemical control was generally achieved in all three cases, we must consider confounding factors. For instance, case 1 had a significant reduction in cortisol level, yet the chemotherapy timeline and ACTH decline support cortisol reduction attribution to chemotherapy’s direct action on the tumor as opposed to metyrapone action alone.

Surgical intervention—tumor resection or bilateral adrenalectomy to address hypercortisolemia—was not pursued in any patient due to the degree of metastatic disease, poor functional status, or patient preference [11, 12]. Despite improvements in cortisol levels and stabilization of metabolic derangements, all three patients ultimately transitioned to comfort‐focused care, which underscores the often palliative nature of ECS management and reinforces the need for early goals of care discussions. These cases reflect real‐world data, in which ECS occurs with advanced, incurable malignancy, and treatment often shifts toward symptom control, as opposed to curative intent [13].

The heterogeneity of presentations, rarity of the condition, and often aggressive tumor biology challenge the ability to develop a standardized pharmacologic approach for the management of ECS. More data are needed to guide optimal therapy selection, dosing, and titration.

## Author Contributions

All authors made individual contributions to authorship and were involved in the manuscript submission. Sachin K. Majumdar was involved in the diagnosis and management of the patients.

## Funding

No public or commercial funding was received for this manuscript.

## Disclosure

All authors have read, reviewed, and approved the final version of the manuscript. Spyridon Ntelis had full access to all of the data in this study and takes complete responsibility for the integrity of the data and the accuracy of the data analysis.

## Consent

As per institution policy, requirement for patient informed consent was waived.

## Conflicts of Interest

The authors declare no conflicts of interest.

## Data Availability

The data that support the findings of this study are available upon request from the corresponding author. The data are not publicly available due to privacy or ethical restrictions.
